# Involvement of the Iron Regulatory Protein from *Eisenia andrei* Earthworms in the Regulation of Cellular Iron Homeostasis

**DOI:** 10.1371/journal.pone.0109900

**Published:** 2014-10-03

**Authors:** Petra Procházková, František Škanta, Radka Roubalová, Marcela Šilerová, Jiří Dvořák, Martin Bilej

**Affiliations:** Laboratory of Cellular and Molecular Immunology, Institute of Microbiology of the Academy of Sciences of the Czech Republic, v. v. i., Prague 4, Czech Republic; Uppsala University, Sweden

## Abstract

Iron homeostasis in cells is regulated by iron regulatory proteins (IRPs) that exist in different organisms. IRPs are cytosolic proteins that bind to iron-responsive elements (IREs) of the 5′- or 3′-untranslated regions (UTR) of mRNAs that encode many proteins involved in iron metabolism. In this study, we have cloned and described a new regulatory protein belonging to the family of IRPs from the earthworm *Eisenia andrei* (EaIRP). The earthworm IRE site in 5′-UTR of ferritin mRNA most likely folds into a secondary structure that differs from the conventional IRE structures of ferritin due to the absence of a typically unpaired cytosine that participates in protein binding. Prepared recombinant EaIRP and proteins from mammalian liver extracts are able to bind both mammalian and *Eisenia* IRE structures of ferritin mRNA, although the affinity of the rEaIRP/*Eisenia* IRE structure is rather low. This result suggests the possible contribution of a conventional IRE structure. When IRP is supplemented with a Fe-S cluster, it can function as a cytosolic aconitase. Cellular cytosolic and mitochondrial fractions, as well as recombinant EaIRP, exhibit aconitase activity that can be abolished by the action of oxygen radicals. The highest expression of *EaIRP* was detected in parts of the digestive tract. We can assume that earthworms may possess an IRE/IRP regulatory network as a potential mechanism for maintaining cellular iron homeostasis, although the aconitase function of EaIRP is most likely more relevant.

## Introduction

Iron is an essential element for all living organisms that acts as a cofactor in fundamental biochemical activities within cells. The major intracellular iron-storage protein ferritin plays a key role in iron homeostasis, and it is omnipresent in animals [1], plants [2], fungi [3] and bacteria [4]. Most ferritins have a similar structure and consist of 24 subunits, forming a hollow sphere capable of storing up to 4500 iron atoms as a ferric inorganic complex [5]. In vertebrates, ferritin is composed of two types of polypeptides, heavy (H) and light (L) chains, that are encoded by different genes [6]. The ferritin of *Eisenia andrei* earthworms is more closely related to the vertebrate H-type subunit [7]. Because iron can be oxidized by the production of oxygen-derived radicals from Fe^2+^ to Fe^3+^ making it toxic [8], [9], there is an essential demand for its regulation by iron-binding proteins at the cellular level.

The expression of ferritin is regulated at the post-transcriptional level by the interaction between cytosolic iron regulatory protein (IRP) and the iron-responsive element (IRE), a structural motif within the 5′-untranslated region (UTR) of ferritin mRNAs [10]. Two IRPs have been described in vertebrates so far. Bifunctional IRP1 can either bind an IRE site or function as a cytosolic isoform of aconitase, while IRP2 has only the IRE-binding activity [11]. The conversion of IRP1 between an IRE-binding protein and aconitase is regulated by iron through the folding or dissociation of a [4Fe-4S] cluster [12]–[14]. When the cellular iron supply is limited, IRP1 binds with high affinity as an apoprotein to the IRE at the 5′-UTR of ferritin. Thus, the IRE/IRP1 complex blocks ribosome binding and the subsequent translation of ferritin [15]. Once the level of intracellular iron is sufficient, a [4Fe-4S] cluster in IRP1 is formed. This protein complex has aconitase activity and loses its ability to bind RNA, allowing the translation of ferritin to occur. Aconitases are iron-sulphur enzymes that interconvert citrate to isocitrate through a stereospecific reversible isomerization [16]. Two aconitases that differ in cellular localization have been found in tissue extracts. There is one form in mitochondria where it acts in the conversion during the tricarboxylic acid cycle [17]. The second form is found in the cytoplasm where it creates isocitrate for other synthetic tasks and can also act as an iron regulatory protein [18]. Messenger RNAs that contain IRE motifs at both their 5′- and 3′-UTRs encode proteins involved in iron storage, iron transport, and iron utilization [8] as well as cellular enzymes. Recently, 35 novel mRNAs that bind both mammalian IPR1 and IRP2 were identified using transcriptome-wide identification [19]. Such mRNAs are also regulated by IRPs, though the *in* *vivo* roles of many of these IREs are not established.

IREs are evolutionarily conserved hairpin structures of ∼30 nucleotides [20] which are recognized by regulatory proteins. They form a “CAGUGN” stem-loop and an unpaired C residue or an asymmetric UGC/C bulge/loop commonly found five nucleotides upstream from the loop in the 5′-UTR of ferritin mRNA [21]. IRE structures have been found in many vertebrates and invertebrates, but some of them, including those from *E. andrei*, show certain distinctions. Based on a computer model of the secondary structure of *E. andrei* ferritin IRE, no conventional bulge is created regardless of whether a cytosine is present five nucleotides upstream of the CAGUGN loop. Instead, a bulged uracil is formed as an optimal secondary conformation [7]. Similarly, the crayfish ferritin RNA stem-loop structure contains a bulge of guanine instead of cytosine at the expected position, but it can still bind IRP1 *in* *vitro*. Moreover, an IRP1-like protein isolated from a crayfish hepatopancreas can bind to the IRE site of crayfish ferritin mRNA [22]. Furthermore, the crustacean *Litopanaeus vannamei* [23] and another member of Annelida, *Periserrula leucophryna*, have guanine bulges instead of cytosine bulges in the IRE sequences of their ferritin [24]. The only metazoan species in which IREs have not been identified and IRPs failed to bind to the ferritin mRNAs are *Caenorhabditis elegans* [25] and *Schistosoma mansoni* [26].

Iron regulatory proteins and ferritins have been described and characterized in a wide variety of animal species, including diverse invertebrate species [20]. We believe that the data about iron regulatory proteins in earthworms can enrich knowledge regarding relevant proteins in other animals.

Based on our previous findings that the IRE structure of *E. andrei* ferritin most likely differs from the conventional structure, we investigated the identification and characterization of the corresponding iron regulatory protein and its interaction with ferritin IRE.

## Materials and Methods

### Isolation of coelomocytes and preparation of cytosolic and mitochondrial fractions from coelomocytes and intestinal tissue

To avoid sample contamination during collection of the coelomic fluid (CF), adult *E. andrei* earthworms (*Oligochaeta*, Annelida) were maintained on moist paper towels without food for two days. CF containing free coelomocytes was obtained by puncturing the post-clitellum segments of the coelomic cavity with a Pasteur micropipette. Coelomocytes were isolated by centrifugation (500 g, 10 min, 4°C) and washed twice with modified isotonic PBS (diluted with water 3∶2 v/v, pH 7.3) before being used in further experiments. Approximately 2.0×10^7^ cells from 6 earthworms were incubated in a solution of digitonin (250 mM sucrose, 50 mM HEPES, 0,007% digitonin, pH 7.4) on ice for 5 min. The mixture was centrifuged at 1,800 g for 8 min at 4°C. The pellet containing the mitochondrial fraction was resuspended and the suspension was centrifuged again for washing. The pelleted mitochondrial material was resuspended in a small volume of 50 mM Tris (pH 7.4), 0,2% Triton X-100, vortexed, and after 5 min centrifuged at 8,000 g for 5 min. The supernatant containing mitochondrial proteins was used for further experiments. The cytosolic fraction of the cells (1,800 g supernatant) was recentrifuged at 230,000 g for 20 min at 4°C. The supernatant was centrifuged on a centrifugation concentrator (Amicon Ultra, Millipore) at 5,000 g for 10 min. Approximately 50 mg of intestinal tissue from 3 earthworms was homogenized in buffer containing 250 mM sucrose, 50 mM HEPES, pH 7.4). The tissue debris was removed by centrifugation at 500 g for 5 min. The supernatant was processed in the same way as the cells samples. The protein concentrations of the mitochondrial and cytosolic fractions were estimated using the Bradford assay (BCA kit, Thermo Scientific) and the activity of aconitase was measured as described below.

### RNA isolation, cDNA synthesis, PCR, and rapid amplification of cDNA ends (RACE)

Total RNA was isolated from the coelomocytes of 3 individual earthworms using the TRIZOL reagent (Life Technologies) according to the manufacturer’s protocol. Two micrograms of DNAse I-treated total RNA were reverse-transcribed using Oligo(dT)_12–18_ primer and Superscript II RNase H^−^ Reverse Transcriptase (Life Technologies) and subsequently used in a PCR reaction. A set of degenerate primers, IRP-B1 and IRP-B3, was designed (Table 1) based on a sequence homology with other invertebrate species. The cycling parameters were as follows: 2 min at 94°C, 35 cycles of 30 s at 94°C, 40 s at 50°C and 90 s at 72°C and a final extension for 10 min at 72°C. The PCR product was ligated into the pCR2.1-TOPO cloning vector (Life Technologies) and sequenced. The 5′-end of *EaIRP* cDNA was obtained using the 5′-RACE System (Life Technologies). The reverse transcription was carried out using 1 µg of total RNA and IRP-specific reverse initial primer 5IRP-16 (Table 1), then a homopolymeric Oligo(dC) tail was added by the terminal deoxynucleotidyl transferase to the 3′-end of purified cDNA. A supplied sense abridged anchor primer (AAP) and an antisense IRP-specific primer 5IRP-13 were used in the first PCR, and a sense abridged universal anchor primer (AUAP) with the specific primer 5IRP-14 were used in a subsequent nested PCR (Table 1). Similarly, the 3′-end of *EaIRP* cDNA was obtained using the 3′-RACE System (Life Technologies). Briefly, 1 µg of total RNA was reverse-transcribed using a supplied adapter primer while a universal amplification primer (AP) in combination with the IRP-specific forward primer F1IRP-3R (Table 1) were used in the PCR reaction. In the subsequent PCR reaction, the AP primer and the IRP-specific forward nested primer F2IRP-3R were used (Table 1). To obtain the entire cDNA sequence of *EaIRP*, four other RACE reactions were performed with primers 8IRP, 7IRP, 13IRP-3, and 14IRP-3. Both 3′- and 5′-RACE products were cloned into pCR2.1-TOPO and sequenced.

**Table 1:** Primers used in PCR, qPCR, cloning and *in* *vitro* transcription.

Degenerate and specific primers used in PCR and qPCR				
type of primer	name	direction	5′-sequence-3′	position nn.
degenerate	IRP-B1	forward	GGNATHGTNCAYCARGT	529–545
	IRP-B3	reverse	CCNARNCCRTTDATCAT	634–650
5′-RACE initial	5IRP-16	reverse	AATATGACCCGTGCCAGATAC	555–575
5′-RACE - PCR	5IRP-13	reverse	ATGACCCGTGCCAGATACTC	553–572
5′-RACE - nested	5IRP-14	reverse	GACCCGGGTGGCACAATCAG	508–527
3′-RACE - PCR	F1IRP-3R	forward	TGCATCAGGTCAATCTAGAGTATC	536–559
3′-RACE - nested	F2IRP-3R	forward	AGAGTATCTGGCACGGGTCATATT	552–575
3′-RACE - PCR	8IRP	forward	GTGGGAGTGGTCGGTAAGTTCGTT	817–840
3′-RACE - nested	7IRP	forward	AGTACGGTGCCACAGTCGGATTCT	911–934
3′-RACE - PCR	13IRP-3	forward	TGTATTTGATGCGGCCGAGAAGTA	2259–2282
3′-RACE - nested	14IRP-3	forward	GAGAAGTACGAAGCGGATGGTCG	2275–2297
qPCR	2RTIRP	forward	CACTGCTGCCCGCTATCTCACTTC	2076–2099
	RTIRP2	reverse	CTTCTCGGCCGCATCAAATACATC	2257–2280
house keeping genes	RPL17for	forward	GCAGAATTCAAGGGACTGGA	
	RPL17rev	reverse	CTCCTTCTCGGACAGGATGA	
	RPL13for	forward	CACAATTGGAATTGCTGTCG	
	RPL13rev	reverse	GTGGCATCACCCTTGTTAGG	
**Primers used for cloning into pRSET B**				
**name**	**direction**	**5**′**-sequence-3**′		
1EaIRPif	forward	ATGACGATAAGGATCCTATGGTTCAGACCAATCCGTTCC		
EaIRPif1	reverse	TCTCGAGCTCGGATCCTCAGAGCAGCTGTCGAATCATG		
**Primers used for ** ***in*** ***vitro*** ** transcription**				
**name**	**5**′**-sequence-3**′			
primerIRE	*TAATACGACTCACTATAG*			
EAconsIRE	TTAGCTCGCACGCACACTGACGCAGCAAAGCACCC*TATAGTGAGTCGTATTA*			
MAMconsIRE	GGGTTCCGTCCAAGCACTGTTGAAGCAGGAAACCC*TATAGTGAGTCGTATTA*			
EAantiIRE	TGCTTTGCTGCGTCAGTGTGCGTGCGAGCTAACCC*TATAGTGAGTCGTATTA*			

### Sequencing and structural analysis

Isolated and purified plasmid DNA was sequenced and the nucleotide sequence of E. andrei IRP (EaIRP) was submitted to the GenBank with accession number JQ407017. The deduced amino acid sequence of the E. andrei IRP and IRP molecules of other invertebrates were aligned using the ClustalW multiple sequence alignment program [27]. The protein sequence was analyzed using Expasy by ProtParam tool [28] for the prediction of the molecular mass and the pI of the E. andrei IRP. Putative conserved domains and binding sites were detected using NCBI-CDD [29] based on related sequences with known structures. The Mfold program was used for the design and comparison of the secondary structure of ferritin 5′-UTR [30].

### Phylogenetic analysis

The IRP sequence from *E. andrei* was combined with all the IRP genes available in GenBank. The dataset consisted of 12 sequences of animal IRPs together with a single representative IRP each for plants (*Arabidopsis*) and protozoans (*Plasmodium*) (Table 2). The amino acid sequence dataset was aligned using the MUSCLE software [31] and gap-containing sites were deleted. The final alignment consisted of 858 positions, from which 299 were conserved and 365 were parsimony-informative. The phylogenetic relatedness was inferred by using maximum likelihood method based on the JTT-matrix-based model using 1,000 bootstrap replicates and default settings in MEGA5 [32].

**Table 2:** The table showing the percentage identities of amino acid sequences of aligned IRPs and aconitases from different organisms.

GenBank ID	Species							Sequence identities (%)									
		*Hs*		*Mm*		*Rn*	*Gg*	*Ss*	*Dr*	*At*	*Pf*	*Dm*	*Aa*	*Ms*	*Pl*	*Ce*	*Ea*
**NP_002188**	*Homo sapiens IRP-1*	100		93		93	88	83	82	59	53	67	68	68	69	64	67
**CAA43455**	*Mus musculus IREBP*			100		96	88	83	83	59	53	68	69	68	71	63	67
**AAA41449**	*Rattus norvegicus IREBP*					100	87	83	82	59	53	68	68	68	69	64	67
**BAA03715**	*Gallus gallus IREBP*						100	83	83	60	54	68	68	69	70	63	67
**ACI33729**	*Salmo salar IREBP-1*							100	88	60	54	67	68	68	68	63	69
**AAZ30732**	*Danio rerio IRP-1*								100	60	53	67	67	68	69	64	68
**CAA58046**	*Arabidopsis thaliana Aconitase*									100	53	58	59	62	60	60	63
**CAB41452**	*Plasmodium falciparum IRP-like*										100	53	53	53	53	56	54
**NP_477371**	*Drosophila melanogaster IRP-1A*											100	76	73	69	64	67
**AAR15297**	*Aedes aegypti IRP*												100	76	69	69	68
**AAK39637**	*Manduca sexta IRP-1*													100	70	65	69
**CAB41634**	*Pacifastacus leniusculus IRP-1-like*														100	63	70
**NP_509898**	*Caenorhabditis elegans Aconitase 1*															100	63
**AFI44047**	*Eisenia andrei IRP*																100

### Tissue expression profile analysis of *EaIRP*

Coelomocytes and various tissues, including the epidermis, seminal vesicles, pharynx, esophagus, crop, gizzard and intestine were collected from at least three adult animals. The total RNA was isolated and reverse transcribed and the obtained cDNA served as a template for qPCR analysis with the iQ5 Real-Time PCR detection system (BioRad) using iQ SYBR Green Supermix (BioRad). Each reaction was performed in a volume of 25 µl which contained 4 µl of the cDNA sample (1∶20 dilution) and 1 µl of primers (0.1 mM 2RTIRP/RTIRP2, RPL17for/RPL17rev and RPL13for/RPL13rev – Table 1). Controls without template were included in all of the experiments. The cycling conditions were as follows: 3 min template denaturation step at 95°C followed by 40 cycles of 30 s at 94°C, 40 s at 60°C and 70 s at 72°C and a final extension for 10 min at 72°C. The temperature was gradually increased to 95°C to obtain the melting curve of the amplified fragments. To be sure that the linear amplification of the template was achieved across a range of concentrations, standard curves for all primers were performed. Quantitative measurements were normalized using the mRNA levels of the *E. andrei* housekeeping genes ribosomal protein 17 and ribosomal protein 13. The gene expression was determined relative to the expression in the epidermis. The values are the means of three experiments (± SD) performed in duplicate, and in each experiment, all parameters were measured in duplicate. The data were expressed as the mean ± SD of the values obtained in all three experiments. One-way ANOVA with Dunnett’s post-test was performed using GraphPad Prism software to evaluate the significance of the data. Differences were considered significant when P<0.05, 0.001.

### Expression, purification and folding of recombinant IRP

The pRSET B-*EaIRP* construct coding for the iron regulatory protein of *E. andrei* was prepared by the In-Fusion cloning system (Clontech) using primers 1EaIRPif/EaIPRif1 carrying BamH1 at the 5′- and 3′-sites (Table 1). In addition to the sequence of *EaIRP*, 6 histidines encoded by the vector are present at the N-terminal end of the protein. For the production of the recombinant protein, the construct was transformed into the BL21 Star (DE3) strain of *E. coli*. Transformed bacterial cells were grown in LB medium with 100 mM ampicillin and 35 mM chloramphenicol in a shaker-incubator at 37°C until OD_600_ 0.4–0.5 and then 1 mM IPTG was added to induce the expression of the recombinant protein. After induction, cells were grown for 6 h and harvested by centrifugation. The cell pellet from 1 l of bacterial culture was resuspended in 20 ml of buffer containing 50 mM Tris-HCl (pH 7.4), 25% sucrose (w/v), 1 mM EDTA, 1 mM NaN_3_, 1 mM PMSF, 1 µM leupeptin, and 1 µM pepstatin. The cell suspension was subjected to 4 cycles of freeze/thaw at −80°C and 37°C and lysed by sonication on ice for 2 min. Disrupted cells were supplemented with DNAse I (100 µg MBU) and RNAse I (100 µg) and incubated at 37°C for 20 min. Following centrifugation at 15,000 g for 10 min, the pellet was washed with 20 ml of buffer containing 50 mM Tris-HCl (pH 7.4), 100 mM NaCl, 1 mM 2-mercaptoethanol, 1 mM NaN_3_, 1 mM PMSF, 1 µM leupeptin, 1 µM pepstatin, and 0.5% Triton X-100 (v/v). The pellet was then washed once again with the same buffer, omitting Triton. After the final centrifugation (15,000 g for 10 min), the washed inclusion bodies were solubilized in 8 ml of solution containing 6 M guanidine-HCl (pH 8), 50 mM Tris-HCl, 100 mM DTT, 1 µM leupeptin, and 1 µM pepstatin and incubated at 40°C for 1 h. The insoluble residues were removed by centrifugation at 20,000 g for 30 min. The supernatant containing rEaIRP was loaded onto Ni-NTA agarose (Machery-Nagel) to purify the protein through binding the polyhistidine tag of the protein to immobilized Ni^2+^ ions followed by elution with 500 mM imidazole. Purified rEaIRP was then refolded by rapid dilution into a 100-fold excess (800 ml) of refolding buffer containing 50 mM Tris-HCl (pH 8.5), 1 M *_L_*-arginine, 1 mM NaN_3_, 1 mM PMSF, 9 mM cysteamine, and 3 mM cystamine and incubated at 4°C for 2 h. The refolding mixture was then dialyzed twice at 4°C against 8 l of 15 mM Tris-HCl (pH 8.5), 9 mM NaCl and 1 mM NaN_3_ for 8 h. A diluted sample was concentrated using centrifugal filter tubes with a cut-off of 50 kDa [33].

### *In vitro* transcription

Putative *Eisenia* IRE-RNA (EAconsIRE), mammalian consensus IRE-RNA (MAMconsIRE, corresponding to the human ferritin H sequence), and a stem-loop negative control RNA (EAantiIRE) sequence were prepared using the MEGAshortscript T7 kit (Life Technologies). IRE primers along with oligonucleotides, EAconsIRE, MAMconsIRE and EAantiIRE to form T7 promoters, were used as templates for *in* *vitro* transcription (Table 1, promoter region italicized). The formation of the double-stranded sequence of the T7 promoter was prepared by heating the appropriate primers (10 µM) together at 95°C for 3 min followed by cooling at room temperature. For the transcription reactions, 1 µM DNA templates and 30 mM Bio-11-UTP (Life Technologies) were used with the remaining non-labeled nucleotides (75 mM dATP, dCTP, dGTP; 45 mM dUTP). Following incubation for 4 h at 37°C, DNase was added and the reaction was incubated for another 30 min at 37°C. The resulting RNAs were purified by phenol/chloroform extraction and alcohol precipitation. The purity and concentration of the synthesized RNAs were analyzed by spectroscopy. The RNA was folded prior to use by heating to 95°C for 5 min, followed by renaturation on ice for 15 min.

### Electromobility shift assay

To prove an interaction between IRE-RNA and IRP, an electromobility shift assay was performed (LightShift Chemiluminescent RNA EMSA Kit, Thermo Scientific). The assay reaction mixtures were prepared by mixing 50 nM rEaIRP or control cytosolic liver extract (4 µg, provided by the RNA EMSA Kit) with different sequences of 6 nM biotinylated IREs (EAconsIRE, MAMconsIRE,) and preincubating the reaction mixture for 30 min at room temperature. For proper folding, all RNAs were heated at 85°C for 5 min followed by cooling on ice for 5 min. Recombinant IRP was reduced using 1 mM 2-mercaptoethanol before the addition of the probe. For competitive experiments, a 200-fold molar excess of unlabeled RNA was added to the reactions. Unlabeled RNAs were prepared as described previously without the use of biotinylated dUTP. Transfer RNA was added to each reaction to act as a nonspecific competitor RNA. The affinity of rIRP for binding to IRE was determined through experiments with constant a concentration of RNA (6 nM) while rIRP was varied from 5–150 nM, or through experiments with a constant concentration of rIRP (50 nM) while RNA concentration was varied from 3–48 nM. The specificity of the binding between RNA and rIRP was assessed through the competitive experiments when different concentrations of unlabeled specific EAconsIRE or unlabeled mutant EAantiIRE probes were added to the reactions (6 nM, 60 nM, 600 nM, 6 µM). The binding reactions (20 µl) containing more binding buffer, 5% glycerol and tRNA (2 µg) were incubated for 30 min at room temperature and separated on 6% native polyacrylamide gels in 0.5X TBE buffer. The gels were run for approximately 4 h at 100 V with 60 min of pre-electrophoresis at 4°C. Samples were then transferred to the nylon membrane using a semi-dry transfer apparatus, and transferred RNA was cross-linked to the membrane by exposure to a UV-light cross-linking instrument with a 254 nm bulb for 1 min. The detection of biotin-labelled RNA was performed by chemiluminescence and exposure to an X-ray film (Kodak) according to the manufacturer’s instructions.

### Aconitase assay

An Aconitase Assay Kit (Cayman Chemical) was used to determine the aconitase activity of rEaIRP in cytosolic and mitochondrial fractions of coelomocytes and intestinal tissue cells. The purity of isolated fractions was confirmed by Western blot with antibodies directed against the cytosolic GADPH and mitochondrial SOD2 (data not shown). The changes in aconitase activity after the addition of 100 µM H_2_O_2_ and a subsequent 1 h incubation were followed. Samples were diluted to a concentration of 1 mg/ml of the total protein and the assay was subsequently performed according to the manufacturer’s protocol. The samples were activated by adding a solution containing 2.5 mM cysteine hydrochloride and 25 µM ferrous ammonium sulfate followed by incubation at 37°C for 15 min in the dark. Aconitase activity was measured using a microplate reader (Tecan) once every minute at 340 nm for 15 min at 37°C. As a positive control, a porcine heart aconitase provided in the Aconitase Assay Kit was used. The activity was determined to be a change in absorbance over time. The following formula for the calculation of the aconitase activity was used: Aconitase activity (nmol/min/ml) = [ΔA/min.(sample) – ΔA/min.(sample+inhibitor)/0.00313 µM]×[0.205 ml/0.05 ml]×sample dilution. The reaction rate at 340 nm was determined using the NADPH extinction coefficient of 0.00313 µM. The values are the means of three experiments +/− SD. A Bonferroni post-test was performed to evaluate the significance of the aconitase changes after incubation with H_2_O_2._ Differences were considered significant when P<0.1, 0.05, 0.001.

## Results

### Sequence characterization

**Figure 1:**
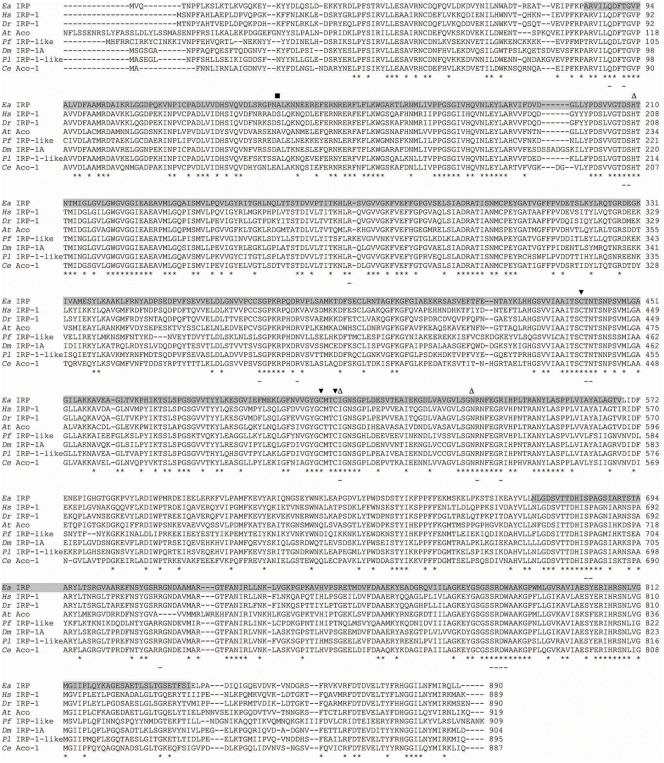
Amino acid sequence alignments of for various IRPs. The alignment of the predicted amino acid sequence of *E. andrei* IRP with *H. sapiens* IRP-1, *D. rerio* IRP-1, *A. thaliana* aconitase, *P. falciparum* IRP-like protein, *D. melanogaster* IRP-1A, *P. leniusculus* IRP-1-like protein and *C. elegans* aconitase-1 using the ClustalW multiple sequence alignment program. Putative conserved domains and binding sites were detected by NCBI-CDD. Two conserved domains of IRP are in gray: (Arg84-Val568: Aconitase catalytic domain, Asn672-Ile839: Aconitase swivel domain). The aconitase catalytic domain includes a ligand binding site that binds to the Fe-S cluster required for the activity. The aconitase swivel domain includes a substrate binding site with residues participating in the active site of the catalytic domain. Asterisks show homology in amino acids in all aligned proteins. Black arrows (▾) indicate three cysteine residues binding the Fe-S cluster. Further ligand binding sites are indicated by white arrows (Δ). Putative amino acid residues involved in RNA binding are underlined (based on NCBI-CDD prediction or from the comparison with human IRP1). The square (▪) shows the position of a serine that can be phosphorylated.

Based on the sequences of the described IRPs of other invertebrates, degenerate primers were designed and used in the PCR reactions. An obtained short sequence was used for the design of specific primers. To assemble the full-length cDNA sequence of *EaIRP*, RACE amplifications of the 5′- and 3′-cDNA ends were performed. The resulting PCR products were cloned and sequenced. Analysis of both the nucleotide and the deduced amino acid sequence of the earthworm IRP identified it as a member of the highly conserved family of iron regulatory proteins. The nucleotide sequence of *EaIRP* has been deposited in the GenBank database under GenBank accession number JQ407017. The full-length cDNA of *EaIRP* comprises 3,187 nucleotides. The sequence contains a 40-nucleotide 5′-UTR followed by an open reading frame coding for 890 amino acids and a 475-nucleotide 3′-UTR containing a putative polyadenylation signal with a poly(A) tail at the 3′-end. The predicted molecular mass of EaIRP is 98 kDa with a pI 6.15. EaIRP is comprised of two conserved domains, which are the aconitase catalytic domain (Arg84-Val568) and the aconitase swivel domain (Asn672-Ile839). The catalytic domain includes ligand-binding sites with three cysteine residues (Cys439, Cys505 and Cys508) that bind the Fe-S cluster. The swivel domain includes the substrate binding sites with residues participating in the active site of the catalytic domain. The amino acid residues involved in RNA binding are present in both domains (Fig. 1). The amino acid sequence of EaIRP has a high similarity to those of other animals (Table 2).

As shown, the earthworm IRP is most similar to the *Pacifastacus* IRP1-like protein (70%) but the identity with other IRPs was approximately equal (63–69%). The smallest homology was found in comparison with a *Plasmodium falciparum* IRP-like protein, which does not belong to animals but is a representative of protozoans. Nevertheless, 67% of the identity of the earthworm IRP with mammalian IRPs suggests a very close relationship and common ancestral origin within this iron regulatory protein group.

### Molecular phylogenetic relatedness

**Figure 2:**
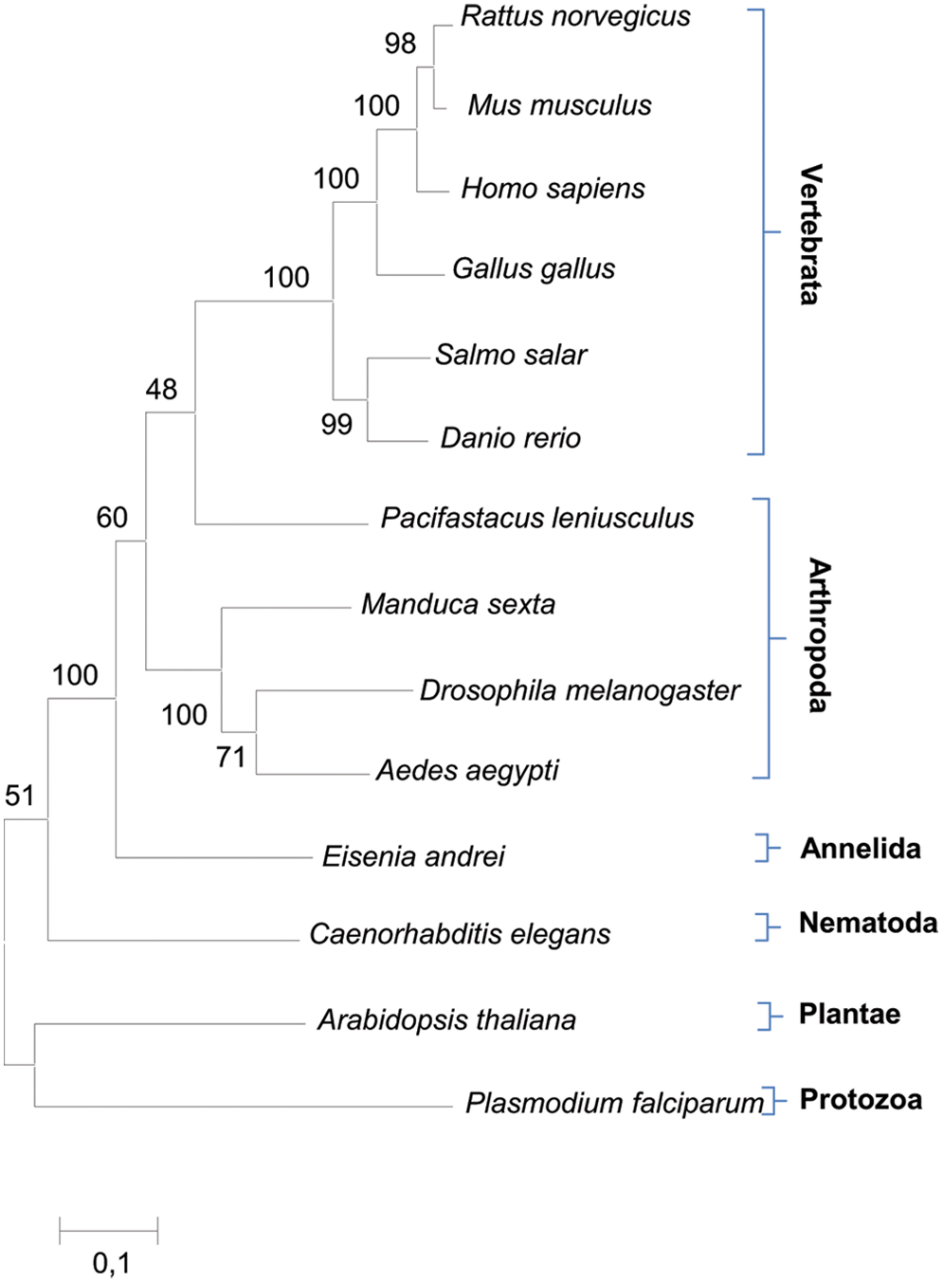
Molecular phylogenetic relatedness of 14 animal IRP genes inferred by maximum likelihood method. The tree is unrooted. The *E. andrei* sequence formed a lineage basal to the arthropoda and vertebrata groups.

The phylogenetic analysis showed the ancestral position of *Caenorhabditis* to the clade consisting of the *E. andrei* IRP gene as well as arthropod and vertebrate genes. Our *E. andrei* sequence formed a lineage basal to these two groups of animals (Fig. 2).

### Cell and tissue expression of *EaIRP*

**Figure 3:**
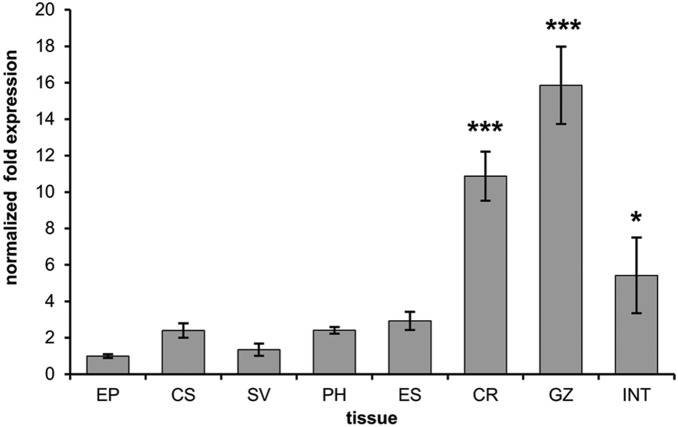
The expression of *Ea*IRP in different tissues. The expression of *EaIRP* in coelomocytes and different tissues was normalized to two different housekeeping genes: RPL13, RPL17. *EaIRP* was constitutively expressed in coelomocytes and in all tested tissues (EP – epidermis, CS – coelomocytes, SV – seminal vesicles, PH – pharynx, ES – esophagus, CR – crop, GZ – gizzard, INT – intestine). The expression was related to the expression in the epidermis, where the expression of *Ea*IRP was the lowest. The highest level of *EaIRP* expression was found in the part of the digestive tract that included the crop, gizzard and intestine. One-way ANOVA with Dunnett’s post-test was performed, using GraphPad Prism software to evaluate the significance of the data. Differences were considered significant when P<0.05, 0.001.

To investigate the tissue expression profile of *EaIRP*, qPCR was performed on various tissues and coelomocytes. As shown in Fig. 3, *EaIRP* was constitutively expressed in cells and in all of the following tested tissues: epidermis, seminal vesicles, pharynx, esophagus, crop, gizzard and intestine. The highest level of expression was in the crop, gizzard and intestine, which form the main part of the digestive tract. The expression was related to the expression in the epidermis where the expression of *EaIRP* was the lowest.

### Expression of recombinant *EaIRP*

**Figure 4:**
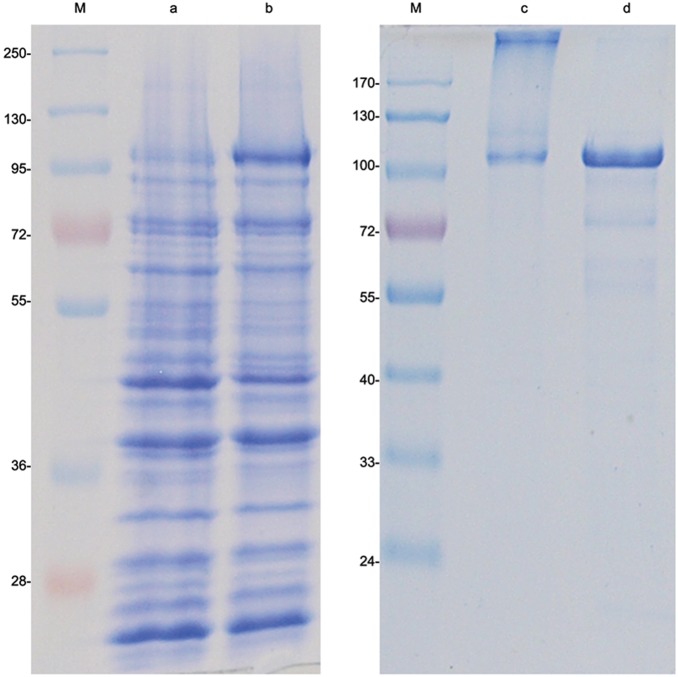
Coomassie staining of SDS-PAGE of *E. coli* lysate proteins transformed with pRSET B-*EaIRP* and purified rEaIRP. Lanes: M – MW markers, a - transformed *E. coli* BL21, b - transformed *E. coli* BL21 induced with 1 mM IPTG, c – purified rEaIRP, d- purified rEaIRP reduced with 1 mM 2-mercaptoethanol.

Using the *E. coli* expression system, we obtained recombinant EaIRP (rEaIRP) in a soluble form that was applied in the following studies. BL21 Star cells grown at 37°C produced rEaIRP in the inclusion bodies after induction with IPTG (Fig. 4). The induction of protein expression under lower temperatures (20 or 25°C) to achieve the production of soluble rEaIRP was unsuccessful. The imidazole eluate from the Ni^2+^ affinity column contained full-length rEaIRP. Due to the presence of protein in the inclusion bodies, the denaturation of the protein followed by refolding was necessary. The folding by dialysis against the decreasing concentration of urea for several days resulted in misfolding of the protein, followed by precipitation. The correctly folded rEaIRP was achieved by rapid dilution into a 100-fold excess of refolding buffer containing arginine, an enhancer of protein refolding, as well as reducing and oxidizing agents to allow for the shuffling of disulphide bonds. The presence of properly formed disulphide bonds was proved by the different electrophoretic profile of rEaIRP after the use of the reduction agent 2-mercaptoethanol (Fig. 4).

### IRE/IRP interaction

**Figure 5:**
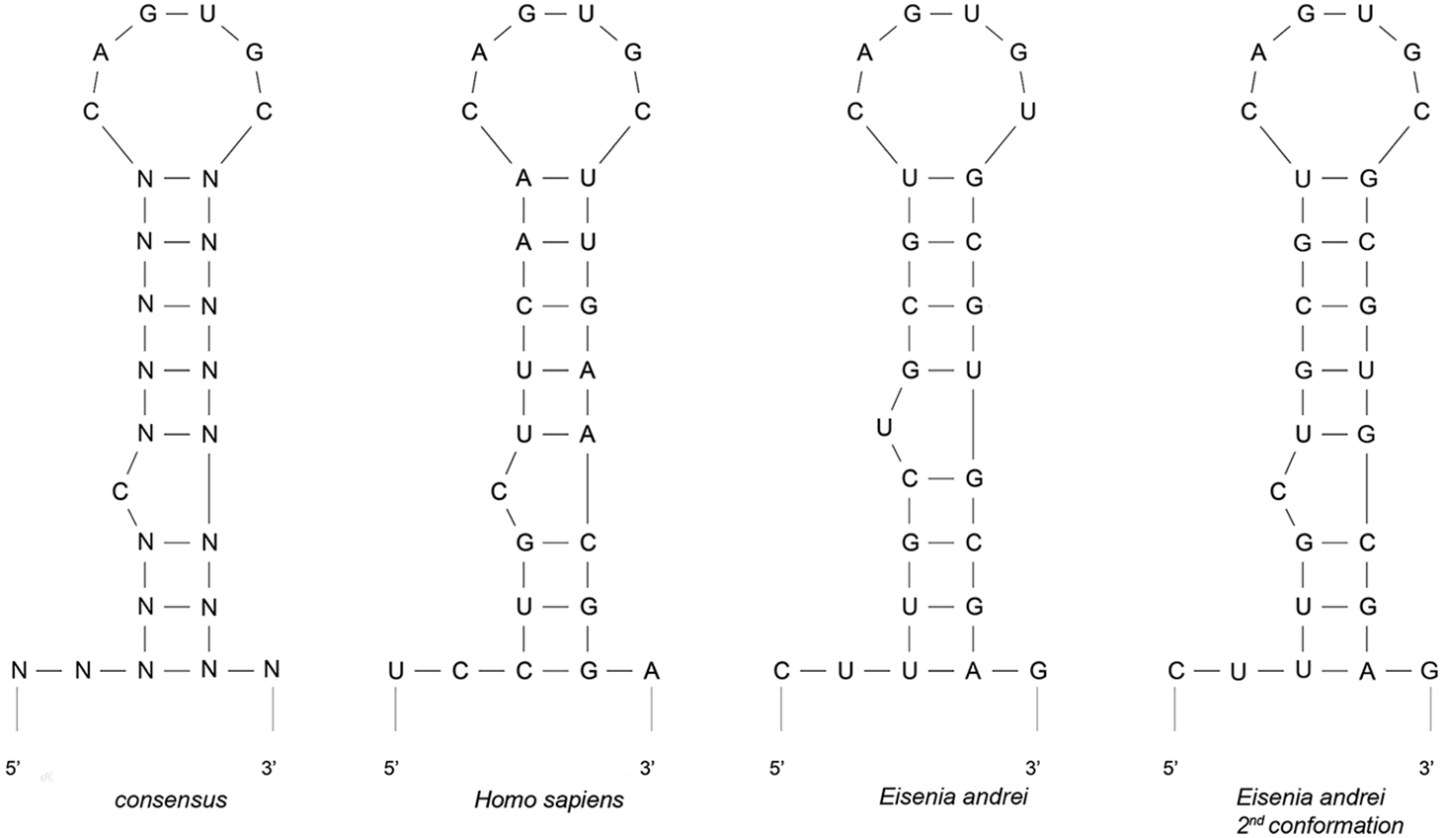
Comparison of the predicted secondary structure of the 5′-UTR sequence of the consensus ferritin IRE to the ferritins of *H. sapiens* and two possible structures of *E. Andrei.* The Mfold program was used for the design and comparison of the secondary structure of ferritin 5′-UTR. The EaIRE site of earthworm ferritin mRNA creates an unpaired uracil one nucleotide closer to the loop as an optimal secondary conformation instead of the conventional bulged cytosine of the typical IRE. The conventional structure with a bulged cytosine can be formed as well, but with very low probability (0.9% calculated by the Sfold server).

**Figure 6:**
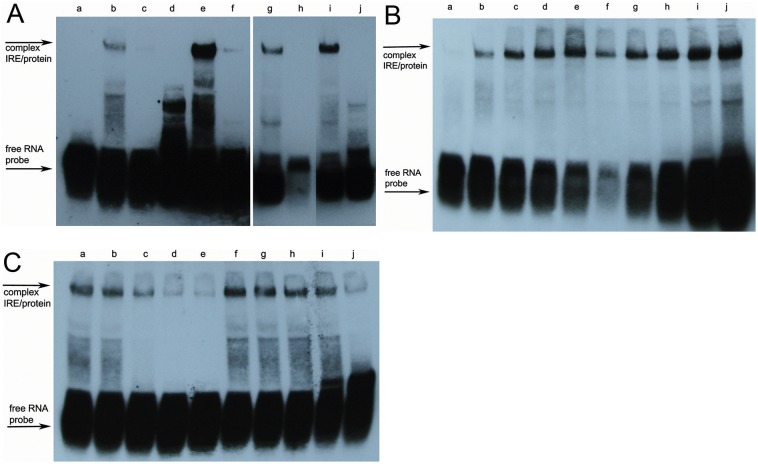
Interaction of ferritin IREs and purified rEaIRP or mammalian liver extract in REMSA. A/Putative biotin-labeled earthworm and mammalian IREs (6 nM) were incubated with either 50 nM rEaIRP or 4 µg of mammalian liver extract and the protein/IRE complexes were resolved on a 6% native polyacrylamide gel. For competitive experiments, a 200-fold molar excess of unlabeled IRE was premixed with the probe before the protein wad added. The positions of the IRE/IRP-specific complexes and of the excess free IRE probe are indicated by arrows. Lanes: a – free EAconsIRE, b – EAconsIRE + liver extract, c – EAconsIRE + liver extract + unlabeled EAconsIRE, d – free MAMconsIRE, e – MAMconsIRE + liver extract, f – MAMconsIRE + liver extract + unlabeled MAMconsIRE, g – MAMconsIRE + rEaIRP, h – MAMconsIRE + rEaIRP + unlabeled MAMconsIRE, i – EAconsIRE + rEaIRP, j – EAconsIRE + rEaIRP + unlabeled EAconsIRE. Labeled RNA corresponding to the IRE site of earthworm ferritin mRNA can be bound by purified rEaIRP and by proteins form the mammalian liver extract, albeit with weaker intensity. The mammalian IRE site of ferritin is also bound by rEaIRP as well as by mammalian liver extract. B/Binding affinity. The rEaIRP concentration was varied (a–5 nM, b–20 nM, c–50 nM, d–100 nM, e–150 nM) with the EAconsIRE present at 6 nM or with keeping the rEaIRP at a constant concentration (50 nM) while the concentration of EAconsIRE was varied (f–3 nM, g–6 nM, h–12 nM, i–24 nM, j–48 nM). A distinct complex was visible when the concentration of rIRP was 50 nM which corresponded to an 8.3-fold of RNA concentration, suggesting a rather small amount of active protein. C/Competitive experiments. To the complexes of EAconsIRE/rEaIRP (6 nM/50 nM), different concentrations of unlabeled EAconsIRE (a – no competitor, b–6 nM, c–60 nM, d–600 nM, e–6 µM) or unlabeled mutant EAantiIRE (f – no competitor, g–6 nM, h–60 nM, i–600 nM, j–6 µM) were added. When increasing concentrations of unlabeled specific RNA were added to the reaction with labeled RNA, the complex of RNA/rIRP disappeared, confirming the specificity of the binding. The addition of mutant RNA did not influence the formation of the RNA/rIRP complex.

An RNA electromobility shift assay (REMSA) was performed to verify whether EaIRP was able to bind to the putative IRE sites. The EaIRE site contained in the 5′-UTR of earthworm ferritin mRNA creates an unpaired uracil one nucleotide closer to the loop as an optimal secondary conformation instead of the conventional bulged cytosine of the typical IRE site. The conventional structure with bulged cytosine can be formed as well, but with very low probability (Fig. 5). The frequency of such a conformation was calculated to be 0.9% using the Sfold server. The REMSA showed that labeled RNA corresponding to the IRE within the 5′-UTR of earthworm ferritin mRNA can be bound by purified rEaIRP or by proteins from the mammalian liver extract, albeit with weaker intensity (Fig. 6A). For competitive experiments, a 200-fold molar excess of unlabeled IRE was premixed with the probe before the protein was added. The specificity of binding was demonstrated by the ability of unlabeled IRE to compete with the formation of a shifted complex. Similar to the earthworm IRE hairpin, the mammalian IRE site of ferritin was also bound by rEaIRP. In a control reaction, the mammalian liver extract and mammalian conventional IRE hairpin was used. The addition of unlabeled IREs and mammalian IRE to the rEaIRP and to the liver extract again resulted in the apparent reduction in the formation of the IRE/IRP complex (Fig. 6A). The affinity of rIRP for binding to IRE was determined by experiments with a constant concentration of RNA (6 nM) while the concentration of rIRP was varied from 5–150 nM. The IRE/rIRP complex was visible with a 20 nM concentration of rIRP. A distinct complex was visible at a 50 nM concentration of rIRP, corresponding to an 8.3-fold of RNA concentration (Fig. 6B) that suggested a rather small amount of active protein. Experiments in which the concentration of rIRP was held constant at 50 nM while the RNA concentration was varied from 3–48 nM resulted in the formation of the IRE/rIRP complex with a corresponding increasing intensity (Fig. 6B).

The specificity of the binding of RNA (6 nM) to rIRP (500 nM) was assessed by the competitive experiments when different concentrations of unlabeled specific EAconsIRE or unlabeled mutant EAantiIRE probes were added to the reactions (Fig. 6C; 6 nM, 60 nM, 600 nM, 6 µM). The constant concentrations of RNA and rIRP were derived from the most favorable conditions for forming the IRE/rIRP complex. When increasing concentration of unlabeled specific EAconsIRE and labeled EAconsIRE were added to the reaction, the complex of IRE/rIRP disappeared, confirming the specificity of the binding. On the other hand, the adding of unlabeled mutant EAantiIRE did not influence the forming of the IRE/rIRP complex. The slight decrease of complex intensity in the reaction with 6 µM unlabeled RNA is most likely due to the excessive amount of RNA.

### Aconitase activity of cellular fractions and *rEaIRP*

**Figure 7:**
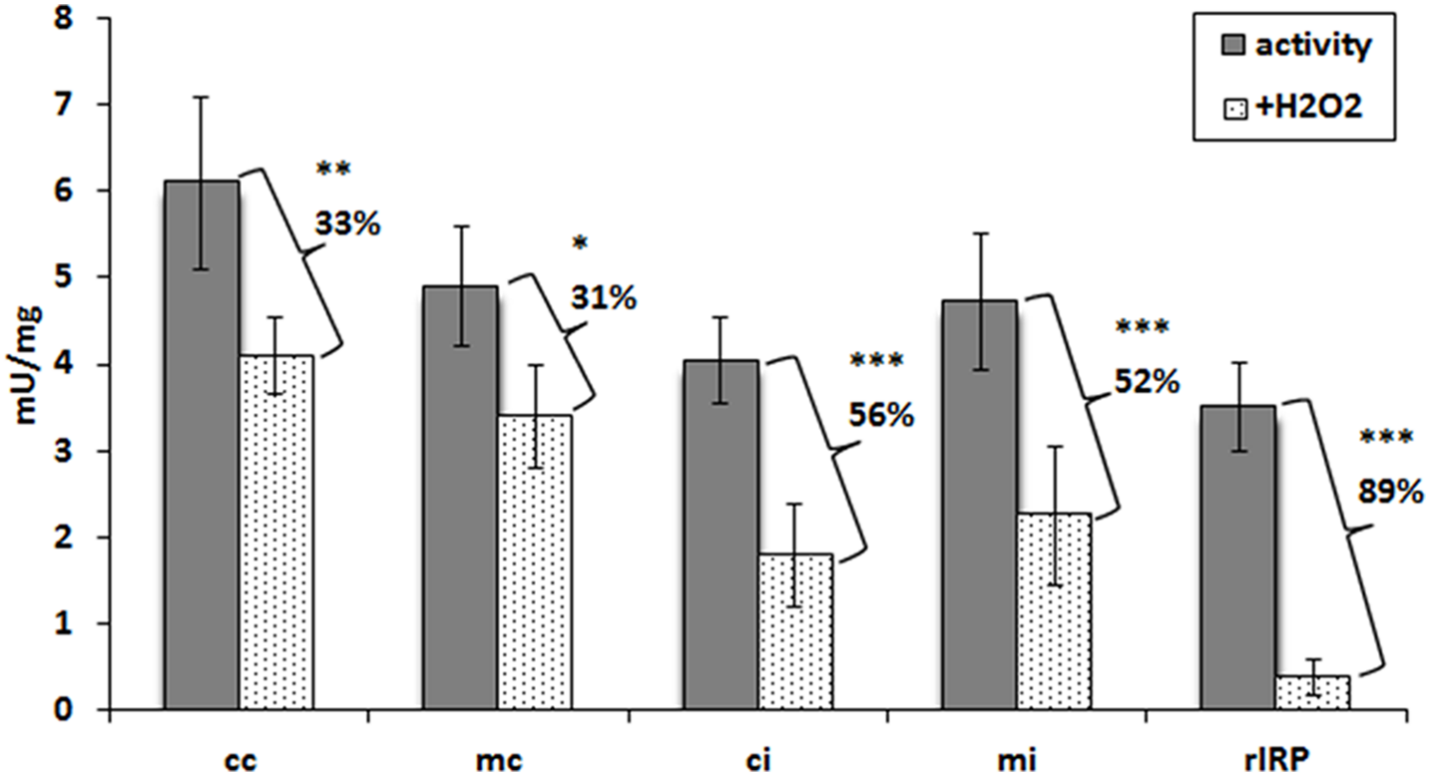
Aconitase activity in cellular fractions of *E. andrei* and rEaIRP. Activity is expressed in milliunits per mg of total extract protein/rEaIRP. The activity was determined in cytosolic and mitochondrial fractions of coelomocytes and intestinal tissue/rEaIRP alone and in the cytosolic and mitochondrial fractions of coelomocytes and intestinal tissue/rEaIRP incubated with 100 µM H_2_O_2_ for 1 h. The values are the means of three experiments ± SD. A Bonferroni post-test was performed to evaluate the significance of aconitase changes after the incubation with H_2_O_2._ Differences were considered significant when P<0.1, 0.05, 0.001. (pa – porcine aconitase used as a control, cc – cytosolic fraction of coelomocytes, mc – mitochondrial fraction of coelomocytes, ci – cytosolic fraction of intestinal tissue, mi – mitochondrial fraction of intestinal tissue, rIRP – recombinant IRP of *E. andrei*). All fractions as well as rEaIRP evinced an aconitase activity, which can be reduced by treatment with H_2_O_2._

A bolus of exogenous H_2_O_2_ elicits IRP activation for RNA binding [34]. The aconitase activity of rEaIRP was assessed in cytosolic and mitochondrial fractions of coelomocytes and in *E. andrei* intestinal cells as well as in corresponding H_2_O_2_ boosted samples (Fig. 7). The activity of samples with an aconitase inhibitor was subtracted from the measured values and was identified as a change in absorbance over time. The obtained activity was thus expressed as milliunits in a milligram of protein. The cytosolic fraction of coelomocytes reached the values of the positive control (porcine heart aconitase provided by a kit, data not shown). Other fractions also evinced an aconitase activity, which could be decreased by the treatment of samples with H_2_O_2._ The reduction of the activity was 31–56% in all fractions. By itself, rEaIRP evinced aconitase activity and incubation with H_2_O_2_ led to a substantial 89% reduction in this activity.

## Discussion

The IRE/IRP interaction represents a unique cellular regulatory mechanism of iron homeostasis in most organisms. This regulatory network functions as a post-transcriptional gene expression regulation system comprising RNA binding proteins (IRPs) and regulatory RNA elements (IREs). Iron regulatory proteins have been described in many organisms including vertebrates, invertebrates, plants and protozoans. Most of these proteins are able to bind to specific IRE sequences. The exception is the *C. elegans* IRP, which shares a resemblance to mammalian IRP1 but fails to bind RNA. This is in agreement with the lack of IREs in the *C. elegans* genome [25]. Similarly, another IRP1-related protein of *A. thaliana* has been reported to be non-RNA binding [35].

Crystallographic analysis of human IRP1 revealed that three iron atoms bind to cysteine residues in the polypeptide backbone (Cys437, Cys503, and Cys506) whereas the fourth one binds to the solvent and interacts with the aconitase substrate citrate [36]. Similarly, EaIRP contains corresponding cysteine residues (Cys439, Cys505, and Cys508) that bind the Fe-S cluster. In humans, the “AGU” pseudotriloop of the IRE hairpin of the ferritin mRNA makes specific bonds with Ser371 (A), Lys379 (G) and Arg269 (U) [36]. The sequence of EaIRP contains Ser373, Arg381 and Arg271 at corresponding places. The substitution of Lys379 with Arg381 is probably not crucial because both are basic amino acids. Other human IRP1 amino acids have been described as important for the binding of the conserved C8 bulge of the IRE stem: Arg713 and Arg780 represent the active site of c-aconitase; Ser681, Pro682, Asp781 and Trp782 form the hydrogen bonds with the base of C8; and Thr438 andAsn439 make direct contact with the IRE [36]. Although all these amino acids are also identically present in EaIRP at corresponding places, the optimal structure of ferritin 5′-UTR does not contain the conventional bulged C8 nucleotide based on the modeling of the secondary structure. It is, however, possible that the conventional structure could be formed as well in a small fraction. Human IRP1 is regulated by the phosphorylation of Ser138 by protein kinase C [37]. The phosphorylation of IRP1 affects aconitase function by destabilizing the Fe-S cluster leading to the accumulation of the RNA binding form [38]. Surprisingly, in EaIRP as well as in IRP from *A. thaliana*, *P. falciparum*, *D. melanogaster*, *P. leniusculus* and *C. elegans* (Fig. 1), this serine is substituted by a nonphosphorylatable alanine. The mutagenesis of IRP1 revealed the loss of aconitase function when Ser138 was mutated to one of the phosphomimetic amino acids aspartate or glutamate. In contrast, the mutagenesis of Ser138 to alanine did not influence the aconitase function of IRP [38]. The presence of alanine in the appropriate place in EaIRP and IRPs of other organisms suggests the absence of the Fe-S cluster stability regulation by phosphorylation.

IRP1 with a bound [4Fe-4S] cluster in the enzymatic active site comes to serve as cytosolic aconitase. Loss of the cluster from aconitase can be induced through oxidation by reactive oxygen species (ROS) or reactive nitrogen species (RNS) leading to the generation of the [3Fe-4S] non-RNA binding form followed by gradual disassembly and complete removal of the cluster [39], [40]. Depending on the cellular iron level, IRP1 could be converted back to c-aconitase or remain in the RNA binding form to regulate IRE-containing mRNAs. Cytosolic and mitochondrial fractions of *E. andrei* cells as well as rEaIRP possess aconitase activity, which can be abolished by treatment with H_2_O_2_ due to the destruction of the Fe-S cluster [8]. Because the aconitase activity of rIRP can be reduced in this manner by 89%, we suppose that EaIRP predominantly acts as aconitase rather than as an iron regulatory protein. IRPs regulates several genes post-transcriptionally, including ferritin [41], transferrin receptor [42], mitochondrial aconitase [43], erythroid aminolevulinic acid synthase (eALAS) [44], ferroportin [45], divalent metal ion transporter (DMT1) [46], succinate dehydrogenase [47], cell division cycle 14a (Cdc14a) [48], and myotonic dystrophy kinase-related Cdc42-binding kinase α (MRCKα) [49]. Depending on the location of the IRE site of the UTR, the regulation of gene expression differs. The evolutionarily conserved hairpin structures of IREs that forms the “CAGUGN” stem-loop and an unpaired C residue or an asymmetric UGC/C bulge/loop, which is commonly found five nucleotides upstream from the loop in the 5′-UTR of ferritin mRNA [21], is considered to be crucial for protein binding. IRE structures have been found in many vertebrates as well as in invertebrates, but some of them, including *E. andrei*, show certain distinctions. Based on computer modeling of the *E. andrei* ferritin IRE secondary structure formation, the presence of a cytosine 5 nucleotides upstream of the CAGUGN loop does not create a conventional bulge. Instead, a bulged uracil is present as an optimal secondary conformation. In our binding experiments, both rEaIRP and proteins from mammalian liver extract were able to bind *Eisenia* or mammalian IRE structures prepared *in* *vitro*. We are aware that multiple conformations of RNAs are always present in the samples and that the conventional conformation with bulged cytosine can be formed as well, but this conformation would be formed with a much lower probability and with a much lower minimum free energy. Because *Eisenia* and mammalian IRE structures differ mainly in the presence or absence of an unpaired cytosine, there are different explanations of how EaIRP binds to RNA. One possible explanation of is that *Eisenia* IRP could tolerate the unpaired residue being located one nucleotide closer to the loop or that only a very small fraction of RNA, which is folded into the structure with a bulged cytosine located five bases upstream, is responsible for the binding of IRP. The second explanation is supported by the fact that the detection of IRE/IRP complexes was performed under conditions allowing quite low affinity interactions. More stringent affinity conditions did not result in the formation of the IRE/IRP complex. Moreover, RNA that remained unbound likely represented incompletely folded RNA or was incapable of binding, which may represent the optimal predicted secondary structure. Because the majority of formed IRE structures potentially do not bind to IRPs, their function in the post-transcriptional regulation is weak.

The cell and tissue expression analysis of EaIRP revealed the constitutive expression in all tested samples with the highest gene expression in parts of the digestive tract. This is in agreement with the fact that EaIRP is an important enzyme affecting many basic cellular biochemical processes. EaIRP has a very high homology with the IRPs of other organisms (Table 2), suggesting a close relationship and common ancestral origin within the iron regulatory protein group. The position of *Eisenia* in the analysis of phylogenetic relatedness (Fig. 2) correlates with the phylogenetic “tree of life” describing the evolutionary relationships between species.

We can assume that earthworms may possess an IRE/IRP regulatory network as a mechanism for maintaining cellular iron homeostasis. However, the aconitase function of EaIRP is most likely more relevant.
